# Reimagining the machine learning life cycle to improve educational outcomes of students

**DOI:** 10.1073/pnas.2204781120

**Published:** 2023-02-24

**Authors:** Lydia T. Liu, Serena Wang, Tolani Britton, Rediet Abebe

**Affiliations:** ^a^Department of Electrical Engineering and Computer Sciences, University of California, Berkeley, CA 94709; ^b^Berkeley School of Education, University of California, Berkeley, CA 94704; ^c^Harvard Society of Fellows, Cambridge, MA 02138

**Keywords:** machine learning for social good, problem formulation, education technologies, education interventions, algorithmic fairness

## Abstract

Machine learning (ML) techniques are increasingly prevalent in education, from their use in predicting student dropout to assisting in university admissions and facilitating the rise of massive open online courses (MOOCs). Given the rapid growth of these novel uses, there is a pressing need to investigate how ML techniques support long-standing education principles and goals. In this work, we shed light on this complex landscape drawing on qualitative insights from interviews with education experts. These interviews comprise in-depth evaluations of ML for education (ML4Ed) papers published in preeminent applied ML conferences over the past decade. Our central research goal is to critically examine how the stated or implied education and societal objectives of these papers are aligned with the ML problems they tackle. That is, to what extent does the technical problem formulation, objectives, approach, and interpretation of results align with the education problem at hand? We find that a cross-disciplinary gap exists and is particularly salient in two parts of the ML life cycle: the formulation of an ML problem from education goals and the translation of predictions to interventions. We use these insights to propose an extended ML life cycle, which may also apply to the use of ML in other domains. Our work joins a growing number of meta-analytical studies across education and ML research as well as critical analyses of the societal impact of ML. Specifically, it fills a gap between the prevailing technical understanding of machine learning and the perspective of education researchers working with students and in policy.

## Significance

Given the rapid proliferation of machine learning technologies in education, we investigate the extent to which development of Machine learning (ML) technologies supports holistic education principles and goals. We present findings from a cross-disciplinary interview study of education researchers, examining whether and how the stated or implied educational objectives of ML4Ed research papers are aligned with the ML problem formulation, objectives, and interpretation of results. Our findings shed light on two translational challenges: 1) integrating educational goals into the formulation of technical ML problems and 2) translating predictions by ML methods to real-world interventions. We use these insights to propose an extended ML lifecycle, which may apply to the use of ML in other domains.

The widespread use of machine learning (ML) across domains remains controversial, with experts exposing concerns around data curation, relevance, and appropriate use of ML techniques as well as the potential for algorithms to create and amplify inequalities. While wide-spread public conversations around the use of ML are a more recent phenomenon, the computer science community has employed ML approaches widely in tasks such as recommendation systems (1) and speech and image recognition (2–4). More recently, numerous other disciplines have turned toward ML to increase efficiency and improve outcomes. For example, ML algorithms are seeing an increase in use across education. They have been deployed in a variety of ways both at the secondary and postsecondary levels, often with a stated goal of improving student performance. Some of the uses include predicting student dropout at the secondary level (5, 6), evaluating applicants in college and graduate school admissions, and predicting persistence in massive open online courses (MOOCs) (7–9).

Extensive research currently explores the use of machine learning for social good (10–13) or “ML4SG.” Despite a surge in interest in understanding the societal impact of ML, this research faces two prevailing challenges. First, empirical evidence on the long-term effectiveness of ML4SG remains sparse (13). Second, despite the application of ML4SG in consequential applications—such as education, environmental protection, and healthcare—inquiry into what “social good” entails in these contexts and the extent to which ML4SG efforts contribute to the relevant social goals remains nascent (14).

Despite the proliferation of ML4SG research in education, a number of recent algorithmic solutions in education (15–17) have led to negative and disparate outcomes, with students from historically marginalized backgrounds bearing the brunt of the burden. While these instances of harm highlight the importance of interdisciplinary collaboration to evaluate the ethics, equity, and impact of ML techniques in the education domain, for instance, via the scrutiny of data sources (18), surveillance practices (19), and transparency of the ML models (5, 9), they also demonstrate that a gap persists between the intent and impact in ML for education domain applications.

Multidisciplinary research communities in education, including educational data mining (EDM) (20, 21), learning analytics (LA), computer-supported collaborative learning (CSCL) (22), and AI for education (AIED) (23, 24), have been active, with overlapping research interests and philosophical differences described in ref. 25. Surveys of research trends and challenges in these communities (23, 26, 27) have indicated rapid growth in the use of technology in education and consequent research interest. This growth necessitates a deep dive into the technological priorities in applied ML research and development, which is central to the current work. A shared interest in common problem formulations and technologies notwithstanding, Calders et al. (21) suggest that there are differences in background and motivation between education research communities and general ML and data science communities like KDD^*^ (28).

Our work focuses on the cross-disciplinary gaps found within mainstream ML communities and, in particular, the gaps between persons developing new ML methodologies for education within a ML community and education experts who research and evaluate the use of technologies in schools and classrooms. We ask, “How does the technical setup of ML papers (objective, evaluation metrics, modeling techniques) match educational goals and principles?” Prior work conceptualizes “epistemic trespassing” (29), where algorithmic contributions from technical researchers overlook important applied context and critical perspective. We specifically study how such gaps surface, through the expertise of education researchers and practitioners, as machine learning researchers formulate prediction tasks in highly complex education settings.

Many research landscape studies (23, 26) focus on overall research trends in the aforementioned education publication venues. Building on this research, we examine cross-disciplinary research practices from the perspective of ML technology development and focus on contributions to the education domain from broad machine learning and AI conferences such as KDD, AAAI^†^, and IJCAI^‡^, over the past decade. These have an audience of computer and data scientists, whereas education technology conferences tend “toward smaller, more focused conferences and communities” with primarily education researchers (24). While ML and AI conferences have published highly cited ML4SG research relevant to education [such as 15, 30], these articles are not typically included in survey studies (23) published in multidisciplinary education technology venues.

We carry out in-depth semistructured interviews with 15 education researchers and practitioners to critically examine the divide between intent and impact in the existing research literature of ML4SG applied to education (“ML4Ed”). Interviewees have domain backgrounds in higher education policy and K-12 education policy. We include researchers across K-12 and higher education because many of the challenges with ML technology use occur in both settings. The interviews were centered around a discussion of research papers on “AI for social good,” compiled by Shi et al. (12), that are relevant to ML4Ed and represent recent scholarship appearing in mainstream machine learning and AI conferences. Specifically, we explore the formulation of the machine learning task, the role and function of prediction, and whether the intended and realized impacts on students are well aligned.

Prior critical work explored cross-domain literature surveys, such as surveys of AI4SG (12, 14) and of algorithmic harms and bias (31, 32). We focus our investigation on a single application domain—education—and draw on insights from interviews with education experts to answer our research questions on the gap between machine learning research practices and the use of ML technologies in education. In contrast, prior fieldwork-based studies explored the practice of machine learning in industry settings (33, 34). By engaging education researchers in discussions of current work in ML4Ed, we use a cross-disciplinary lens to bring to light facets traditionally overlooked in ML research. While study participants do not directly speak for the lived experiences of stakeholders such as students, teachers, parents, and institutions, we draw upon their nuanced and broad understanding of various stakeholders’ perspectives and interactions with these stakeholders.

While we focus on education as a specific and important domain, many of our findings can extend to other domains and highlight common areas for improvement in machine learning practices. By studying ML communities’ forays into education applications, we tease out both education-specific lessons and general lessons that apply to applications of ML in other domains.

We present two main findings in *Translating Education Goals to ML Problems* and *Translating Predictions to Interventions*), which correspond to the misalignment of machine learning experts and education researchers with respect to the problem formulation and the limits of prediction tasks. For the first finding, narrowing multidimensional outcomes to a single quantifiable metric that can be computed by an algorithm can lead to oversimplification of complex educational problems and the neglect of education equity and access goals. This theme was echoed by numerous educational researchers and practitioners. Our second finding highlights that prediction is useful when it provides actionable information for interventions to improve outcomes but can lead to negative outcomes if prediction is treated as the primary goal. Given these findings, we discuss improvements to machine learning systems and approaches used in education to increase the likelihood of positive educational outcomes for students.

## Materials and Methods

This study is based on data generated from in-depth semistructured interviews with 15 education researchers discussing selected research papers that apply ML to education (“ML4Ed”).

### ML4Ed Papers.

The papers we discussed during the interviews were sampled from a dataset of research papers on “AI for social good” compiled by Shi et al. (12). The dataset contains 1176 papers published between 2008 and 2019, of which 78 papers were labeled as “education”-related by Shi et al. via keyword matching. We then selected papers from this set that were relevant to machine learning based on their abstracts^§^, leading to a list of 20 papers (“ML4Ed list”) (6–8, 15, 30, 35–49). We randomly examined unselected papers and confirmed that there was no significant exclusion of papers pertinent to ML4Ed by our sampling process. Major topics in education that are covered by the final set of papers include higher education, student learning, MOOCs, and standardized assessment. Notable omissions include special education, early education, and teaching.^¶^

### Participants.

Interview participants were recruited through purposive sampling (50, 51) from the authors’ professional networks In order to be invited for our study, participants needed expertise in education research and practice. The current roles of the participants include Ph.D. candidates in education and economics of education, postdoctoral researchers, university faculty members, and research directors at public and private education agencies. In terms of education level, we included a mix of participants with expertise in K-12 and higher education. We refer to them anonymously as P01–P15. *SI Appendix*, Table S1 lists all participants’ self-reported occupations, research areas, genders, and races/ethnicities. In addition to current occupations, Table 1 lists other experiences in the education sector that participants have had. Further details that participants provided on their backgrounds indicated common experiences with teaching in public high schools, engagement in policy evaluation, and nonprofit work focused on students from working class families.

**Table 1.** Education sector experiences that participants have had aside from their listed occupations in *SI Appendix*, Table S1

Education sector experience	# of participants
Taught at a university	10
Created or designed curriculum	9
Built tools used in the education sector	8
Taught at an elementary/middle/high school	7
Worked with policymakers	7
Worked with not-for-profit organization(s) or NGO	6
Worked with for-profit organizations in education	5

### Matching.

Each interview focused on one paper from the aforementioned ML4Ed list. We matched papers to participants by respecting the participant’s preferences. Participants filled out a preinterview survey (Dataset S1), indicating up to six papers that they are “willing to discuss” or that they “prefer to discuss” during the interview. The authors selected one of the participant’s preferred papers for discussion during the interview, prioritizing the coverage of education topics (Dataset S2, *SI Appendix*, Tables S2 and S3 for the list of papers and topics) whenever possible. Finally, nine unique papers were discussed during the interviews.

### Interviews.

Our interview data were generated from December 2020 to September 2021. Interviews lasted between 50 to 60 min and were conducted through Zoom. Participants were sent the relevant ML4Ed paper beforehand for voluntary perusal. We asked participants’ permission to record the interview via a consent form as well as verbally confirmed their consent before beginning to record the interview. Each interview comprised two parts: 1) introductions and background questions and 2) a focused discussion on the research paper. Part 1 included questions on the participant’s current research interests in education and on the impact of data science or machine learning in education contexts that they are familiar with. After contextualizing the participant’s professional experience, we began Part 2 by reading together selected sections of the ML4Ed paper and followed the reading of each section with discussion prompts such as “How would you describe their goals?” and “To what extent do you feel this machine learning task captures the ... goal?”. All interviews were recorded and transcribed.

### Data Analysis.

We analyzed the data inductively. In the initial stage, all four authors conducted open coding on Atlas.TI and met biweekly to compare generated codes and discuss emergent themes. From the codes generated by individual authors, a preliminary list of codes were identified through consensus that included themes such as education goals of machine learning technologies, from machine learning tasks to interventions, problem complexity, and human stakeholder engagement. The authors returned to code selected interviews with the preliminary list of codes, individually, and, in subsequent meetings, discussed and reconciled differences and continued to refine the salient themes. This process was iterated until the authors reached a consensus on the final list of themes and how they were assigned to text. Finally, the first two authors labeled each theme with a short name, wrote up informative definitions, and recoded the dataset in its entirety.

Further details on our data and methods, including summaries of participant information (Dataset S3), interview questions (Dataset S4), and codes (Dataset S5), can be found in the supporting material.

### IRB Approval.

This study was approved by the Committee for Protection of Human Subjects (CPHS), which serves as the institutional review board (IRB) for the University of California, Berkeley.

## An Extended ML Life Cycle

**Fig. 1.**
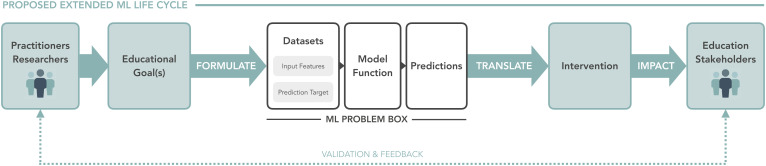
An extended ML life cycle diagram. The inner “ML Problem Box” represents the typical aspects of the ML problem detailed in the surveyed ML research papers. Our interview findings reveal the need to consider an extended version of the ML life cycle in ML research, including the initial problem formulation stage by practitioners and researchers and the translation from predictions to interventions that eventually impact stakeholders.

To frame our findings, we start by providing an overview of the ML life cycle as it pertains to the education papers surveyed. We illustrate this in the extended ML life cycle diagram (Fig. 1) and refer to this as a conceptual tool to capture the key findings from our interview study. We also define the terminology we use for the different parts of the ML process.

Grounding this terminology in the education domain, we consider a running example of predicting student dropout risk, which was a common application among the papers from Shi et al. (12)’s Survey on AI for Social Good (6, 7, 30, 42). In a typical application of supervised machine learning, the practitioner decides on a particular dataset to use for model training and validation (e.g., historical student data over the past several years). The dataset comprises input features, denoted *X* (e.g., student grades, attendance, demographic information), and a prediction target, denoted *Y* (e.g., whether or not a student dropped out of a program). The goal of the practitioner is to find a model function *f*, typically through mathematical optimization, such that the output of *f* when applied to the input features, *f*(*X*), closely approximates *Y*, not only on the dataset but also on new samples. This procedure is referred to as training. *f*(*X*) is said to output predictions for *Y*. ML papers often focus on the family of functions over which *f* is trained, such as regularized linear models, neural networks, and decision trees.

The aforementioned elements make up the ML Problem box pictured in Fig. 1. In the ML4Ed papers surveyed, these elements are typically prominently featured and discussed. Indeed, these elements can have significant ethical implications and impact on outcomes. For instance, sampling and historical bias in the input features and prediction target have been scrutinized in the literature on fairness, accountability, transparency, and ethics (FATE) (31, 52).

However, as much as scrutiny of the ML Problem is crucial, we found that much of the critical discussion on ML4Ed research from our interviews falls outside the traditional ML problem box—in fact, all fifteen interview participants raised discussion points outside of the traditional ML problem box in the following ways. First, before the ML problem is even formulated, practitioners and researchers must identify a set of education goals to address (in the running dropout risk prediction example, these goals may include increasing on-time graduation rates). This influences the choice of input features, prediction target, and success metrics used to evaluate the model function. Once the model function is trained, the subsequent interventions that apply the predictions produced by the trained model function are frequently overlooked in the ML research papers surveyed (in the risk prediction example, who are the dropout risk scores shown to?). Thus, Fig. 1 extends the ML life cycle outside of the ML problem box to include these consequential steps. Mirroring these important aspects of the ML life cycle that may be overlooked by ML4Ed research papers, our research findings are organized around two translational challenges:

1. Translating education goals to ML problems. How education goals are interpreted by computer science researchers and translated into a machine learning problem;
2. Translating predictions to interventions. How the predictions of ML models are “translated” into interventions and how to evaluate and address their impact.

In the following sections, we elaborate on the above findings from our interview study.

## 1. Translating Education Goals to ML Problems

We first discuss issues and recommendations that arise in the process of translating education goals into the ML problems detailed in the ML4Ed papers. Many impactful design decisions arise just in this problem formulation stage, including in the choices of success metrics, targets, and inputs.

### A. Conflating Education Goals with Quantitative, Short-Term Metrics.

While ML4Ed research papers often sought to address education goals, such as student learning and student success, they tended to reflect a narrow understanding of these goals by focusing exclusively on a single quantitative metric. Ten (out of fifteen) interview participants identified limitations in the single quantitative metric highlighted in ML4Ed papers spanning multiple problem contexts, from secondary success and essay scoring to college persistence and graduate admissions. For example, an ML4Ed paper may choose to tackle a specific goal related to a single metric such as “improving on-time graduation rates.” Interviewees pointed out that though the chosen metric, graduation rate, is relevant in the context of student success, there are other metrics that also should be taken into account in order to capture student success (five participants^#^).

P01 (Executive director and Professor): “We don’t limit ourselves to graduation rates. We’re also looking at other things such as retention rates and so forth.”

P02 (Director of research): “Graduating with satisfaction or knowing exactly what they want to do afterwards. That’s another thing that we could look at.”

While both P01 and P02 agree that graduation rate is a useful metric, they each mention other important metrics that are possibly longer term or more difficult to quantify.

#### Lack of substantive justification.

While the single metric might be an important outcome, as in the case of college graduation rates, substantive justification for why metrics matter often seems to be missing, beyond correlation with longer-term student outcomes or the existence of data for the metric. P03, a faculty member in a school of education, observed that in ML4Ed research, “there is often bias toward shorter term outcomes without drawing out the logical map of why do we care” because “there is better data about them [...] they’re more often in the same dataset.” Accounts of why the chosen metric is important may be overly inflated and, more importantly, inaccurate to educational realities.

The focus on one metric also leads to overemphasis on exam scores as the learning metric. Commenting on some ML4Ed research papers’ usage of exam scores as a target, P04 (Senior lecturer) observes, “It just sounds like somebody who doesn’t actually know schools. It sounds like somebody who just sort of like has a theory in mind of how tests matter.” P04 discusses how persons might focus on standardized exam scores without a deep understanding of what they measure and how they are related to student outcomes.

Another example of this focus on exam scores comes from automated scoring of assessments. In the context of student learning, the focus on automated scoring centers on the role of performance on assessments, as opposed to other aspects of learning, such as self-expression and creativity. For example, P05, a PhD candidate in education, finds that an automated essay scoring tool “assumes that there is a universal good way to write.” While automated feedback may be useful to a student, to “help point little things out,” any such tool necessarily encodes “a lot of values [...] that [are] not being made transparent” (P05, PhD candidate). Moreover, the possibility of automating certain forms of assessment through technology warrants serious reflection on the relevance of such assessments and whether they are meaningful for students.

P05 (PhD candidate): “If the writing is so mechanical that it’s very easy for a computer to grade it like a human, then what are we even asking students to do in the first place?”

The value of assessment as an education goal should not be taken for granted, as P05 notes above.

#### Competing goals and stakeholders.

In the context of graduate admissions, participants point out that education institutions can have competing goals. However, ML4Ed research may address one of them without acknowledging the tensions with the others. Driven by “the current business model of higher education,” universities in the United States tend to optimize for “efficiency,” according to P06, a PhD candidate in education. P06 further elaborates, “they want students who would have higher rates of retention or graduation [...] otherwise, that’s a financial loss to them.” However, many, especially public institutions, also have a mandate to promote education equity and access. Focusing almost entirely on making the admissions process “more efficient,” the ML4Ed research paper proposed to use only past admissions decisions to inform their applicant-rating algorithm while neglecting to address the concerns with education equity and access. In fact, if past application processes had led to lower rates of admissions for candidates with diverse academic profiles, an algorithm using this biased decision data likely led to similar admission outcomes.

Other than the institution’s equity goal, the needs of key stakeholders, such as students, are also systematically left out by the selective attention that ML4Ed gives to particular education goals, as quantified by a single or a limited number of metrics, such as time savings. When asked whether a published admissions algorithm would meet educational needs, P07 (Vice president) responded,

P07 (Vice president): “Whose needs? The student’s needs, probably not. If you ask a student, do you want your application that you spend a lot of time on [...] to just go through an algorithm? I think they would say no. If you ask [...] your chief diversity officer, in their opinion, does this work well? They probably want to see, well, who’s being admitted and who’s being not admitted [...] For the faculty, yeah, it’s working well because what they want is to spend less time and get high quality students admitted.”

In this example, the interests of faculty do not converge with either the desire of students to have a holistic review of their applications or the institutional interest in having a more diverse set of strong candidates for admission.

Although a single quantitative metric can contain important information, it rarely captures the rich tapestry of student experience or the varied institutional objectives. Instead, the choice to focus on one or few metrics reflects ML4Ed’s unexamined preferences for short-term and easily quantifiable education goals. In the following section, we delve deeper into the assumptions behind the choice of a single metric as the prediction target.

### B. Consequential Choices in Problem Formulation: Target and Input.

In the formulation of a supervised machine learning problem^ǁ^, the choice of a prediction target and input features is paramount. Yet the value-laden nature of these choices is rarely examined in ML4Ed research. Discussions with study participants suggest that expedient choices in problem formulation can have unintended implications for the research project and its applications down the road.

#### Universalizing individual narratives with prediction target.

In the education context, any choice of prediction target will inevitably lead to a loss of important information—the qualitative aspects of individual experience and narratives. Data that cannot be quantified in the prediction target are effectively dropped by a machine learning model. The irreducibility of qualitative perspectives is particularly salient in the automated evaluation of student writing. P05 (PhD candidate) worries about outweighing these with a “zoomed out computational perspective.”

P05 (PhD candidate): “There’s a lot of sociolinguistic variation in these essays. [...] You’re asking students to write about themselves and [...] we have these unique, interesting experiences. And of course, they’re going to be reflected in the essays.”

Similarly, in the context of graduate admissions, P06 (PhD candidate) stressed the importance of reconstructing individual narratives, as opposed to relying on quantitative summaries.

P06 (PhD candidate): “I think holistic admission is not like putting all these characteristics into a prediction model and then see[ing] what’s the probability. [...] You’re taking it into account these different pieces of information [...] to restore the actual life experience [...] difficulties they overcome throughout their life experience to achieve, to arrive where they are.”

Both participants express reservations that “blunt force” (P05) algorithmic solutions may not adequately summarize the variation and unique narratives that are central to the holistic evaluation of student essays.

In these settings, participants noted that ML4Ed researchers picked a single prediction target and evaluated individuals accordingly, thus demonstrating a “universal[ist]” attitude. According to P05 (PhD candidate), not only is ranking “the best essays” a misdirected way to think about the students’ life stories, such an approach also assumes that the same metric applies to everyone.

P05 (PhD candidate): “Clearly the ideas of what’s good writing in English are not transportable [...] in general, that’s not a universal thing and these kinds of platforms, I think they’re kind of built and designed with universality in mind, [that] any student can use this.”

P05 noted that formulating a machine learning problem with universality in mind runs the risk of discounting the rich and nuanced experiences of low-income students and students of color in particular, who may be underrepresented in the data.

#### Alternative problem formulations.

Problem formulations that do not rely on a single prediction target may be better aligned with the needs of the stakeholders. In the setting of graduate admissions, P06 (PhD candidate) questioned whether instead of giving “an explicit ranking”—based on “whether a student, given their characteristics, would [have been] admitted or not in the past”—the algorithmic system could “give summary information to the officers [...] to reduce the workload or [...] cognitive load,” which is “even more aligned with people’s initial demand.” P06 suggests using ML technologies to provide multiple dimensions of information on applicants. In this way, they avoid using biased past application data while potentially providing information on applicant strengths and areas of growth.

#### Alignment of prediction target with education goals.

Even in cases where a single prediction target is appropriate, researchers might not end up selecting one that is well aligned with education goals. When predicting test outcomes, for example, the choice of test is important. One ML4Ed paper that sought to identify secondary students “at risk” of “poor academic performance” based its prediction target on student performance in two exams: a state test and a national standardized test.

P04: “[A general standardized test] more just measures like the opportunities they’ve had in their life in general as opposed to the curriculum they were supposedly getting in school [...] you were saying like they had more opportunity or more privilege in their background than actually measuring if they studied the material or if they learned.”

Here, P04 (Senior lecturer) questioned whether these tests directly measured a student’s mastery of the specific content of their secondary curriculum and, if not, whether what they were measuring fit with the project’s education goals.

Another common practice in problem formulation is to convert a measurement, such as a test score, to a binary prediction target using an arbitrary threshold. In the context of identifying students who are academically at risk, this conflates students who are struggling academically with students who are just below the cutoff. P08 (PhD candidate) stressed that “those are different groups,” alluding to the “bubble kids phenomenon” in educational testing (53). Other participants expressed concern that this focuses attention unnecessarily on passing a particular assessment, however arbitrarily this may be defined.

P04 (Senior lecturer): “Maybe the assessment is one piece of evidence among many that you might use to make that decision. But this person or the authors are making it sound like if you don’t pass the test, you don’t progress.”

Given the often arbitrary thresholds, basing decisions on how students will progress or the interventions that they might receive on prediction targets with arbitrary thresholds could lead to more negative outcomes for students.

#### Expedient input features.

Apart from the choice of a prediction target, the choice of input data features is also significant. In several ML4Ed projects, participants including P12 (Assistant professor) found that this choice is dominated by convenience and data availability considerations.

P12 (Assistant professor): “[The input features are] just the stuff that we happen to be able to look at. It’s not clear at all that this is what you would really want to know about a human being to figure out whether or not they were having a meaningful learning experience in a course.”

Commenting on student data collected from MOOCs, P12 opined that “these systems are not designed from the beginning to instrument things,” and the data features that are easily available do not necessarily capture meaningful properties.

This inadvertently leads to a narrow and, sometimes, overly individualistic view of student experience that downplays social and structural issues. For example, when machine learning systems are trained on academic and curricular data alone, socioeconomic factors such as student finances and family are left out of the bigger picture of student success. Commenting on an ML4Ed paper on college student performance prediction, P02 (Director of research) says she “feel[s] a little uncomfortable thinking that graduation time and graduation grade is entirely dependent on course work which is really not true for college.” Instead, there are institutional decisions such as how much aid students receive each semester and macroeconomic conditions that could induce changes to familial financial circumstances. The models do not account for factors that relate to institutional choices that change over time and impact the likelihood of student success; yet nonmalleable factors related to student race are taken into account. In the following section, we examine the use of demographic information as input features, within the broader context of education equity.

### C. Designing Inputs with Education Equity in Mind.

Educational access and equity are primary social goods. While several participants emphasized the importance of acknowledging and addressing structural inequality in education-related research, the ML4Ed papers rarely explained how their research understands existing education inequities. ML technologies that are agnostic to structural inequality, such as the systematic disadvantages faced by underresourced institutions, can actually widen gaps in access to quality education (*SI Appendix*, section 1.B).

#### Unconsidered use of race data for prediction.

Structural inequality manifests as disparities in educational opportunity and advantage across demographic groups. When it comes to ML4Ed research, however, the use of demographic information should be approached with care, according to several participants.

P04 (Senior lecturer): “[We] would be sending the wrong message if we said, we were controlling for race and ethnicity, because it might imply that we thought that kids who were Black or Hispanic [...] were less likely to succeed. ”

Commenting on a paper that included race as an input feature for a machine learning model that predicts student academic performance, P04 warns that this choice has unexamined normative implications, such as sending the “wrong message about how we assess risk.”

Even if the inclusion of attributes led to increased predictive accuracy (when measured on a particular dataset), such nominal improvements must be weighed against how the data are used and interpreted, and the broader implications of the decision to explicitly include, and therefore, reify, race as an input to the predictor. Importantly, including race and ethnicity, without accounting for the ways in which resources and opportunity are allocated by race and ethnicity, can lead to erroneous conclusions about the role of race and ethnicity in outcomes. This decision sets a standard for how race is understood and instrumentalized in that education context—which can be problematic. In the following excerpt, P04 contrasts a state agency’s ultimate decision to exclude race from their predictive model with the ML4Ed paper’s approach.

P04 (Senior lecturer): “[Race] was a little too nuanced and it wasn’t adding enough predictive validity to make it worth the cost of the potential for people being up in arms about the state’s way of doing these models. [...] But a researcher would never think of it that way, right? They [...] want to get the best prediction possible, and you probably would add a little bit to your predictive ability if you had that in there.”

If the researcher considers only the predictive power of the race feature without thinking about the social implications of including it, this may lead to negative consequences when the model is used.

Even if race were included as an input to a model, it can potentially have a large measurement error. To illustrate one of the many complications, P14 (Postdoctoral researcher) points out that there can be “different race and ethnicity variables across the different datasets;” not all demographic information that agencies “collect” is self-reported. Self-reported racial and ethnic data could significantly diverge from data collected by government agencies.

We discuss the limitations of using demographic information to audit a machine learning model for its impact on educational access in *SI Appendix*, section 1*E*.

By giving consideration to education equity from the beginning—when formulating education goals into ML problems—researchers can work to address the real and pressing problems of equity and access in education. In all, problem formulation choices in ML4Ed are often underdetermined, yet many have far-reaching consequences.

## 2. Translating Predictions to Interventions

We have shown how effective translation from education goals to problem formulation choices can be challenging, especially in settings with multiple stakeholders and long-term goals. However, the ML life cycle does not end here—we next discuss how thinking critically about interventions and impact can further guide key design decisions and research directions.

### A. The Gap between Predictions and Interventions.

One of the most common and consequential gaps that participants identified in the ML4Ed papers is the gap between prediction tasks and real-world interventions.

#### Predictive accuracy is no panacea.

Instead of focusing on interventions, most machine learning papers tout improvements in prediction accuracy—that is, how often the predictions turn out to be “correct,” by some measure—whether it is predicting student dropout risk, predicting test question difficulty, or predicting likelihood of admission. However, improvements in prediction accuracy do not automatically translate into improved outcomes and, in fact, may not affect outcomes at all. This gap surfaced in the majority of interviews (12 out of 15).

P12 (Assistant professor): “[Researchers] are trying to make tiny, substantively completely irrelevant improvements in the area under the curve of some prediction algorithm, ‘can we predict 62% instead of 61%,’ but nobody had anything to do that would actually help these people.”

P13 (Full professor): “You don’t improve things by predicting them better. There is a missing link there obviously between how we act on predictions in social spaces that are incredibly complicated to improve outcomes.”

In the risk prediction setting, P09 (Assistant professor) and P08 (PhD candidate) warn that even if a prediction of student dropout risk is highly accurate, without adequate resources to help those students, this information ultimately does not benefit institutions.

P09 (Assistant professor): “Even if you tell [schools] [...] that [a student has] a 97% chance of dropping out based on our training data, that’s a difficult thing to take in especially in the public schools [where it is] very difficult to find good teachers for those students.”

P08 (PhD candidate): “A lot is put on schools, a lot is put on teachers. [...] An unfunded mandate on teachers and what they are expected to do in the classroom [with a risk prediction tool] could be bad.”

Thus, the “accuracy” of risk prediction is not a driving force for improving student outcomes, as it does not provide a means to improve student outcomes.

#### Validation within an intervention pipeline.

If predictions do not directly translate into interventions, what is the value of improving them? Supposing that there is eventually an intervention in mind and that the prediction is a concrete component of the intervention pipeline, then P13 (Full professor) comments that improvements in prediction quality can lead to incremental improvements in the efficacy of the known, larger intervention pipeline. Mistakes to avoid, however, include “overselling” the contributions of the ML component or forgetting that additional data-driven methods will be required to validate the full intervention pipeline.

Six participants highlight the importance of validation in the translation from prediction to interventions. P13 (Full professor) emphasizes that education practitioners will “ultimately still have to test down the line” the impact of the intervention. Similarly, P02 (Director of research) comments that “the next step after prediction” is a causal inference problem testing the efficacy of the downstream intervention. To even begin to measure the effect size of an intervention, “you need to have an intervention in mind” (P02, Director of research). While causal A/B testing is common practice in industrial applications of ML, many ML4Ed papers do not reference this need.

#### Forward-looking policy recommendations.

As ML researchers explore new techniques, intervention pipelines that utilize these new technologies may not yet be well established. If the intervention pipeline is not established, can such research and development of predictive models still add value? Perhaps this is possible, especially if ML papers include downstream consideration of interventions and policy recommendations, a practice that is standard in, for example, quantitative subfields of economics. After all, when ML papers already reference policy-driven motivations such as improving student financial situations, it would be remiss not to include a discussion of downstream interventions stemming from this motivation, as P02 suggests below.

P02 (Director of research): “It’s not about just predicting and identifying who is going to be at risk...we should think about, ‘is the financial aid program that we’re offering the right thing to do or do we need to make changes there?”’

Devising new intervention pipelines and policy recommendations that effectively bridge the gap between prediction and intervention is likely to require further interdisciplinary collaboration between education practitioners and ML researchers.

### B. Harms of Naive Translation from Prediction to Intervention.

When ML researchers do not engage with education experts to thoroughly consider interventions, this can lead to naive application of predictive models that cause unintentional harm. In this section, we focus on the topic of risk prediction and leave discussions related to automated grading and tutoring to *SI Appendix*, section 1*A*.

#### Potential harms of student risk scores.

According to five participants, the translation of predictions to interventions is a particularly salient issue in early warning systems applications for high schools, higher education, and MOOCs, where ML4Ed papers proposed new models for predicting individual student risk, for example, of dropping out of a course or not graduating on time. A “failure” event easily translates into a concrete binary outcome, making it a common prediction target for ML papers.

Unfortunately, the ease of quantifying the target does not directly translate into effective interventions. P12 (Assistant professor) points out that one naive assumption that ML papers tend to make, either implicitly or explicitly, is that “showing people more data is good.” Some ML papers claim that they may “[present] at risk students with meaningful probabilities of failure.” However, the efficacy of sharing risk probabilities with individuals is not grounded in behavioral research.

P12 (Assistant professor): “The odds that [presenting at risk students with probabilities of failure] is going to be helpful for students just seem so phenomenally low to me. Have the authors of this paper...seen any evidence talking with students who are in a kind of marginal state? [...] It doesn’t seem well connected to research about what motivates people.”

In fact, directly showing risk scores to students, teachers, or administrators can worsen student outcomes. If a system categorizes students as “at risk,” then P01 (Executive director and Professor) points out that “there is a tendency for a lot of these systems to stigmatize students.” Such stigma from a “deficit-minded” classification can demotivate students (P10, Senior director).

Even if a system is only implicitly categorizing a student as at risk, students are sensitive to differential treatment and cues alluding to their abilities.

P06 (PhD candidate): “If a tutoring algorithm systematically underestimates female students’ mastery levels and provides them with instructional sequences or feedback messages for struggling students, some female students might question their own abilities which could decrease their motivation. Eventually this might lead to a self-fulfilling prophecy.”

#### Institutional level harm.

In addition to influencing students’ views of themselves, naive application of student risk scores can also negatively influence the students’ support structures, including teachers, parents, and institutions. If shown risk scores directly without any additional guidance, a teacher “might allocate more of their limited time to other students rather than a student that the model seems to predict that they will not graduate” (P09, Assistant professor). Similarly, “a parent might stop investing in that child or spending as much time with them” (P09). If risk scores are shown at the institutional level, this can lead to allocation of resources that align more with institutional incentives than student well-being.

P08 (PhD candidate): “If schools [focus] too much on what [they] think students can achieve, [they] end up putting in these artificial barriers and filtering [students] in ways that [they] think are going to work for them or more pessimistically work for the school.”

At the institutional level, P08 (PhD candidate) further points out the potential for additional “surveillance of the students who are at risk,” possibly without the student’s knowledge or consent. Thus, students may not be comfortable with the knowledge that their data are used for risk prediction. P01 (Executive director and Professor) expresses similar reservations even if the risk scores are withheld.

P01 (Executive director and Professor): “If we had an ordinal ranking of students by risk and so forth, I would not be comfortable with sharing that with students nor would I be comfortable with saying, ‘we have it but we’re not going to tell you.’ ”

The publication of ML models for risk prediction can endorse naive interventions with risk scores if those same papers do not discuss the downstream usage and potential harms. The responsible and beneficent use of risk scores in the education sphere is still an open problem for future ML4Ed research.

### C. Toward More Intervention-Aware Predictions.

We now outline ways that prediction tasks can be better formulated with interventions in mind. This includes participants’ existing success stories and suggestions for future work.

#### C.1. Need for actionability (vs. interpretability).

To mitigate harms from the naive announcement of predicted risk scores, participants repeatedly pointed to the value of building models that provide actionable insights, where “actionable” generally refers to the ability to take helpful actions. In the risk prediction setting, P10 (Senior director) recommends stepping away from the “deficit language” and “focusing on the ways in which the student can move forward positively, and hopefully get to graduation.”

P10 (Senior director): “We don’t have to say, ‘You’re not going to succeed.’ We can say, ‘Let’s talk about what are the decisions that you need to make, what is the pathway forward that will allow you to succeed.’ ”

P03 (Associate professor) notes that the risk score is a blunt instrument that students cannot directly use to improve outcomes.

P03 (Associate professor): “ ‘You’re in the 10th percentile for something’ sounds different than ‘we’re worried because you’ve been absent a lot.’ ”

Actionable insights on the path toward success, such as telling a student that the absences may hurt their class performance, can lead to clearer interventions.

While many ML papers included analysis of feature importance in their risk prediction models, this version of interpretability—making a machine learning model more understandable to a human (54, 55)—fell short of addressing experts’ needs in providing actionability. P04 (Senior lecturer) notes that feature importance analysis of nonmutable traits such as demographics is not always useful for developing interventions and may distract from the analysis of more actionable behavioral factors.

P04 (Senior lecturer): “What is most interesting about it to me is not, ‘I wonder if the demographic factors matter more than the behavioral factors.’ To me it’s more about, ‘what can we actually do to help kids get off the trajectory they’re on if they’re not on a good trajectory.’ ”

P04 further joins two other participants in pointing to causal evidence as a useful tool for showing the value of behavior changes. We discuss the connection to causal inference further in *Actionability and Causality*.

#### C.2. Design for empowered human operators.

In tandem with actionable insights, five participants highlighted the importance of designing ML to empower human operators such as academic advisors, teachers, administrators, and admissions officers. Going beyond simple corrective actions, these operators may use ML tools to amplify their ability to achieve broader education goals.

##### Centering the role of teachers.

In a classroom setting where the teacher is a central human operator, predictive models can improve the teacher’s capacity to provide individual student attention and reduce their workload. For instance, automated tutoring tools can effectively reduce the burden on the teacher as a distributing authority for information.

P11 (Associate professor): “First you look online or you ask a friend; if that doesn’t help, then you ask another friend; if that doesn’t help, then you go to the teacher and so that way the teacher is sort of able to distribute themselves a little more evenly.”

When the teachers have the power and resources to intervene, risk prediction algorithms can also “help [teachers] catch some kids that maybe had fallen below the radar before and giving them another source of data on that” (P04, Senior lecturer). Key to this statement is that the algorithm is just acting as “another source of data” in tandem with the teacher. The goal of identifying students that the teachers would otherwise have overlooked is different from the standard goal of achieving high accuracy for the whole population of students. This suggests that the value of risk prediction systems would be improved if they were designed with the teacher’s partnership in mind.

##### Centering the role of academic advisors.

In addition to teachers, participants highlighted academic advisors as key human operators that ML systems can aid. When it comes to student advising, instead of thinking, “this technology will solve our problem,” P10 (Senior director) prefers the attitude, “this technology will be a tool in our toolkit while we do our job that will [...] hopefully have a positive impact on students.”

A key situation when an ML system falls short alone but works well in partnership with a human advisor is when there is ambiguity in the student’s needs and students “don’t know what they don’t know.” Effective usage of automated chatbots or search engines requires knowing what to query; however, P03 (Associate professor) states that “where students get held up is not knowing the questions that they need to ask.”

P03 (Associate professor) “If I don’t know the specific terminology that’s used at my school, or if I don’t know how to think about like a certain question, or if I can articulate my goal but I don’t have a knowledge of all the different paths that could get me to that goal, I think that’s where [...] technology driven advising solutions can’t advise students as well.”

Instead, in between the specific questions that students can ask chatbots or search engines, advisors are “able to fill in the gaps for students” to help them “envision a pathway” from a relatively vague conversation about their broader goals. P10 (Senior director) provides a specific example of an advisor guiding a student to such a point when the student provides only vague guidance on their goals:

P10 (Senior director): “Instead of saying, ‘You’re not going to be a nurse, sorry, like, good luck,’ it’s more, ‘Well, [...] we have respiratory therapy, or we have nutrition, or we have bioinformatics. We have all these other healthcare disciplines that might allow you to help people, to work in healthcare, and to get a job, which [are] three boxes you said you wanted to check. And in some cases, the students are like, ‘That sounds great. I had no idea what an occupational therapist even was.’ ”

According to P07 (Vice president), one obstacle to realizing these benefits of pairing technological tools with dedicated advisors and teachers is that “often machine learning is really hidden within an ed-tech tool.” This means that users and administrators alike “don’t understand all the places where [ML] actually is embedded now.” For example, in admissions, a predictive model may be embedded so deeply in the decision pipeline that admissions officers or the higher-level administrators are not fully cognizant of its role. The lack of transparency coupled with limited user technical knowledge makes it difficult for a human operator to audit or modify the usage of ML within these systems, including overriding incorrect predictions or providing feedback to improve the ML models. This underscores the need for ML systems and pipelines that are designed with the empowerment of human operators in mind.

## 3. Discussion and Related Work

Machine learning promises automated procedures that recognize meaningful patterns in education data and provide principled, real-time decision support and interventions to improve educational outcomes (56, 57). However, before any of these promises can be realized, practitioners and researchers must traverse the entire machine learning life cycle (Fig. 1), from goal identification and problem formulation to intervention and impact evaluation. Through our qualitative work bringing the expertise of education researchers to bear on the research practices of ML4Ed, we found that varying levels of attention have been paid to different parts of the life cycle.

In *Translating Education Goals to ML Problems*, we showed that the consequential choices in the translation of education goals to machine learning tasks are currently overlooked. We found that multifaceted education goals are often reduced to a single quantitative metric, while expedient choices in prediction target and input lead to the omission of key education goals, such as education access and equity, and stakeholder interests. In *Translating Predictions to Interventions*, we outline gaps in the translation from prediction tasks to interventions, including negative externalities of naive application of predictive models, and a path forward from education researchers toward formulating more intervention-aware prediction tasks. Taken together, this work contributes insights to the ongoing conversation around machine learning and its impact in education as well as broad, cross-domain critical discussions at the intersection of algorithmic fairness, accountability, transparency, and ethics (FATE) and AI for Social Good (AI4SG). Situating our findings in FATE, and related fields, we discuss both shared insights and tensions that emerge from our work.

### A. Related Work in Education Technology.

The educational data mining (EDM) (20, 21), learning analytics (LA), computer-supported collaborative learning (CSCL) (22), and AI for education (AIED) (23, 24) communities have been active for the last one to three decades. Retrospective surveys of the research trends and ongoing challenges of these fields have analyzed common and growing ML paradigms being applied in education, like neural networks for learning characteristic prediction and teacher evaluation (23, 58), NLP for language education (59), and AI-assisted personalization (23, 60, 61). Our contribution to this discourse is discussing the implications of and gaps in these chosen ML paradigms, with a target audience of ML researchers who are invested in developing improvements on top of these paradigms or novel paradigms entirely.

Based on the findings of this study, we reaffirm and provide further technical commentary on some of the challenges presented by education technology research communities. For instance, the AIED retrospective by Chen et al. (23) highlights the challenge of adoption by human operators and propose “exploring how human and automated instruction can most effectively be combined to best support instruction” as a future research direction. We show this to be a challenge with publications in general ML conferences as well and further propose several ways that the ML research community can approach this issue. In particular, we suggest focusing on actionability and formulating ML problems to empower human operators.

The distinction between prediction and intervention, as well as the challenge of designing interventions with predictive models, has been acknowledged in prior work in AIED and LA, particularly in the context of dropout prediction (62–64) and predictive learning analytics (65–68). Still, many works from the EDM and AIED communities focus on predictive performance (23, 67, 69–71), with less discussion of whether or how the improvement in predictive accuracy translates to better outcomes. A number of recent empirical studies (72, 73) have found the effect sizes of targeted interventions based on student risk predictions to be statistically insignificant. Our work builds upon these observations to further problematize the assumed relationship between “accurate” predictions and beneficent interventions, which, as far as we know, also exists in the education technology community (74).

Recent work in the intersection between education technology and FATE has addressed the impacts of education technology on equity and ethics (27, 75, 76). Madaio et al. (75) apply a critical theory lens to evaluate the impacts of education technology and algorithmic fairness notions on education equity. Holstein et al. (76) analyze ways that “AIED systems” may alleviate or amplify inequalities under current practical usages. Holmes et al. (27) set a framework for the “Ethics of AIED” through a survey of researchers that publish in the journal and conference of Artificial Intelligence in Education. Our work adds to this discourse in three ways. First, we focus more broadly on impact than on ethics and equity per se. Second, instead of interdisciplinary AIED communities, we target technical ML communities that are potentially driven more by algorithmic novelty than societal needs [see related, 77]. Finally, our methodology of studying the disciplinary boundary crossing of ML communities through interviews with education experts provides an additional distinct evidentiary lens.

Given our different audience and broader focus on impact, our study consequently foregrounds a different set of questions and recommendations than the ethical framework of Holmes et al. (27). For example, Holmes et al. (27) discuss the “value of transparency” as motivated by policy but does not mention actionability, which is a more impact-driven desideratum. Holmes et al. (27) also extensively discuss issues of data governance, such as privacy, anonymity, ownership, and control. These critical ethical problems are much better addressed from their interdisciplinary policy lens and transcend the technical ML problem formulation choices that we discuss.

Despite these differences in focus, we also view equity as integral to evaluating choices in problem formulation and interventions, and many of the recommendations from these works align with ours. Madaio et al. (75) note that education AI technologies “are forged in historical relations of power” and “may reproduce structural injustices—regardless of the models’ accuracy or fairness.” Holstein and Doroudi (76) discuss disparities in access and usage of education AI technologies. Both of these effects can lead to the types of gaps between prediction and intervention surfaced in our interviews in *Translating Predictions to Interventions*. Our work further joins Madaio et al. (75) in concluding that “quick technical solutions and neat group-level evaluations of ‘AI fairness’ ” are not enough to produce adequate solutions to complex issues of education equity. The proposed extended ML life cycle (Fig. 1) constitutes our approach for illustrating the limitations of focusing on “neat” technical solutions to equity challenges. Finally, a common recommendation shared by all of these works is the importance of focusing on the human operator: Holstein and Doroudi (76) highlight the importance of AIED systems’ ability to communicate limitations and “hand off control to humans,” and Holmes et al. (27) discuss ethical issues of human agency. Our work adds to this discussion with an angle of providing specific recommendations for how ML researchers can empower human operators through problem formulation choices and development of better-targeted methodologies.

Beyond these discussions of education equity and ethics, our work also connects with other styles of argument in FATE more broadly, which we discuss in the next section.

### B. Related Work in FATE.

Emergent critical scholarship in FATE has pointed out the gap between the abstract goals of computational research and system design and their operationalization, often in a sociotechnical system (32, 33, 78). Past studies have diverse methodological approaches, scopes, and abstractions to guide their critical inquiry (79). Measurement modeling from quantitative social science suggests that many of the harms of computational systems discussed in the FATE literature (80) can be traced to mismatches between “unobservable theoretical constructs” and how they are ultimately reified as measurements (78). Our work finds there to be varying degrees of mismatch between abstract education goals and their operationalization in the extended machine learning life cycle, going beyond the issue of measurement to, for example, question the theoretical constructs themselves. By highlighting the selective interpretation of education goals, our findings suggest that the choice to focus on a single theoretical construct, particularly narrowly defined constructs, such as “strength of candidate” in the context of graduate admissions, already sidelines certain education goals, often, educational access and equity.

Our work echoes a key insight of a recent ethnographic study on the formulation of corporate data science problems that “problem formulation is a negotiated translation” and has normative implications (33). The results in *Translating Education Goals to ML Problems* highlight the normative implications of problem formulation in the education context, where the goals of employing data science and machine learning are arguably more nuanced and multifaceted than in the corporate context and the social stakes higher. By contributing a distinctly interdisciplinary perspective grounded in expert knowledge of the education domain, our work builds on both the critical scholarship and the body of practical guidance for negotiating translational gaps when machine learning is applied to consequential domains.

In terms of practical guidance, a pioneer study on racial bias in health risk prediction surfaced the issue of label bias and advocated for more careful choice of the prediction target as a way to mitigate racial disparities in predictions (i.e., recommending additional care for Black patients at a lower rate than for similarly ill White patients) (52). In our study, participants also pointed to the risk of choosing prediction targets that are actually proxies for immutable socioeconomic and demographic factors. Our findings support paying greater attention to the choice of the prediction target in ML4Ed problem formulation, but the reasons go beyond the mitigation of prediction bias. By highlighting other complications such as the loss of nonquantitative information, the alignment with education goals and needs of stakeholders, and threshold effects, our work provides a broader account of the important factors that should go into the choice of a prediction target.

### C. Connections with HCI.

This broadening of scope and critical reflection of the design process also has a long history in the human–computer interaction (HCI) and science, technology, and society (STS) literature, where concepts of reflective design (81), participatory design (82), and value sensitive design (83, 84) provide frameworks to bridge the gap between designers, users, and their implicitly held values in the design process. Shared by these design frameworks is the idea that a computational system designer’s choices embed implicit assumptions and values, and in order to best serve the user, the system designer must incorporate the evaluation of these values into the development process. Our work brings similar critical reflection to research papers applying ML in education. Just as the system designer’s scope extends beyond implementation to also incorporate the values and needs of the user and societal context, we also find that ML researchers could have a more positive impact in the education sphere if they incorporate critical evaluation of values and interventions into the choices made in problem formulation.

The HCI literature on human-centered algorithm design (85, 86) relates closely to our discussion on the translation from predictions to interventions through human operators and includes case studies from outside of the education domain such as the US criminal justice system (87) and the US child welfare system (86). Human-centered algorithm design goes beyond requiring human oversight of algorithms, which can be limited or ineffective (88). Floridi et al. (14) posit the importance of respecting user autonomy and “optionality” through “receiver-contextualized intervention” in AI for social good projects. We join these works by providing examples and insights grounded in the education domain (e.g., the partnership with the teacher–advisors) that speak to the value of empowering human operators more systematically.

### D. Actionability and Causality.

Our findings around interventions also contrast the clean division between causality and prediction problems drawn by Kleinberg et al. (89). The framework in ref. 89 does not explicitly recognize the translation from prediction to intervention; however, our findings may be used to extend the framework by Kleinberg et al. (89) to more thoroughly evaluate the practical efficacy of prediction tasks (we elaborate on this with an illustration in the *SI Appendix*, section 1*C*).

Emerging work in causal inference suggests ways to develop intervention models from data as opposed to prediction models in various domains, e.g., refs. 90 and 91, but existing causal inference approaches such as randomized controlled trials have known challenges with external validity (92, 93); as such, identifying actionable insights with causal methods remains an ongoing project. Moreover, Kohler et al. (94) and Hu et al. (95) have pointed out significant conceptual flaws with interpreting social categories such as race and gender as causally manipulable variables [such as in a causal diagram (96)], suggesting that the validity of causal inference cannot be taken for granted where demographic aspects of student data are concerned.

## Supplementary Material

Appendix 01 (PDF)

Dataset S01 (PDF)

Dataset S02 (XLSX)

Dataset S03 (PDF)

Dataset S04 (PDF)

Dataset S05 (XLSX)

## Data Availability

Full interview transcripts are confidential per signed agreements with interview participants. All materials used in the data gathering process are available in *SI Appendix*, including interview questions, preinterview survey questions, and titles of papers discussed during interviews. All annotation codes used during data analysis are also available in *SI Appendix*.

## Footnotes

^*^ACM SIGKDD International Conference on Knowledge Discovery and Data Mining.

^†^The AAAI Conference on Artificial Intelligence.

^‡^International Joint Conference on Artificial Intelligence.

^§^We initially filtered the 78 education-related papers by the presence of keywords (“predict” and “machine learning”) in their abstracts. We then finely selected the papers by reading the abstracts of each paper to ensure that the selected paper involved 1) formulating and solving a machine learning problem and 2) an education-related application. The last step was performed using the authors’ respective expertise in machine learning (L.T.L., S.W., and R.A.) and in education (T.B.).

^¶^The education topics were identified by T.B., who works in education research, based on paper titles and abstracts.

^#^P01, P02, P03, P08, and P09.

^ǁ^Supervised learning was most common in the ML4Ed papers discussed.
