# Supplementary material for: Reimagining the machine learning life cycle to improve educational outcomes of students
Source: Proc Natl Acad Sci U S A. 2023 Feb 24;120(9):e2204781120. doi: 10.1073/pnas.2204781120 (PMC9992853; doi:10.1073/pnas.2204781120)
Supplement: Supplementary file 1 — Appendix 01 (PDF) [file pnas.2204781120.sapp.pdf]

Supporting Information for

Reimagining the Machine Learning Life Cycle to Improve Educational Outcomes of Students

Lydia T. Liu, Serena Wang, Tolani Britton, Rediet Abebe

Correspondence to L.T.L. (lydiatliu@berkeley.edu) and S.W. (serenalwang@berkeley.edu)

This PDF file includes:

Supporting text
Fig. S1
Tables S1 to S3
Legends for Dataset S1 to S5
SI References

Other supporting materials for this manuscript include the following:

Datasets S1 to S5

Supporting Information Text

1. Additional findings

This section include additional findings that supplement the contributions in the main text.

A. Limits of automated interventions. One application of machine learning methods to education is powering automated interventions, including MOOCs, intelligent tutoring systems, and chat bots. In these interventions, ML most effectively serves learning goals when applied to highly specific, narrowly scoped, structured tasks. However, for more complex tasks, these automated interventions still struggle without human partnership.

> P03: "Automated 'nudges' are most effective for very specific types of behaviors [...] anything more complex than a one-step process is not easily nudged."

Thus, MOOC designers can "use data to figure out who needs to be contacted and when is the best time to contact," and intervene with automated "nudges" to maintain student engagement. For undergraduates at a large public university [*], a targeted nudge and chat bot system was most effective at encouraging students to solve "specific problems that were very administrative in nature," such as "submitting the FAFSA, paying off your balance, registering for courses, and finding the parking office." However, according to P10, "it didn't have a big impact on motivating students to choose a major faster or making some of these bigger, [...] more significant decisions."

Another automated intervention is the intelligent tutoring system, where ML models are applied to identify student errors and misconceptions. Two participants identified that these automated interventions have been successful in helping students learn specific structured tasks such as solving algebraic equations or correcting spelling and grammar. However, P11 emphasized that the success of this tool in correcting specific algebra errors "doesn't mean it's going to make a kid love math."

While these automated ML-driven interventions alone are not effective at achieving broader learning goals such as motivation and inspiration, they may still serve these ends by fitting into a learning ecosystem composed of teachers, peers, and advisors. For example, the automated feedback that a student receives from an intelligent tutoring system can be part of a multi-pronged approach that includes self-study activities and references such as homework and textbooks in addition to the aforementioned peer, teacher, and advisor support.

> P11: "If they don't understand the feedback, or if the feedback doesn't seem to help them make progress, then they might go to the teacher or they might go to a peer as part of an ecosystem of resources that they can draw from [...] I can see it as part of a balanced breakfast."

One reason why intelligent tutoring systems need to be part of an ecosystem to go beyond encouraging specific behaviors is that broader learning goals are more complex and require more context than the algorithm is equipped to handle.

> P11: "The algorithm doesn't get why [a behavior] is useful. They're not able to communicate to the student why it's useful [...] Reminding people of how the material connects to the activities is really important."

Teachers are thus better equipped to contextualize the behaviors and feedback with outside materials and broader learning goals. The output of ML systems serves as one tool that teachers can use to support students.

As aforementioned, interventions focused on feedback and error correction also cannot replace a full teaching ecosystem because they do not address the underlying need for student inspiration and motivation. P11 points out that these intelligent tutoring systems can't "make kids love math" or address why some kids "don't want to do computer science." Perhaps this also points to a lack of attention in the machine learning community to these tasks of promoting student motivation and inspiration. Under the current state of the art, the additional context and personal interactions from teachers and academic advisors are essential to filling in the learning ecosystem surrounding highly structured ML tasks.

B. Translating predictions to interventions: Automated grading and tutoring. As in risk prediction, predictive models naively applied in automated grading and tutoring systems can also lead to negative externalities and additional opportunity for misuse, especially in under-resourced institutions attempting to maximize limited resources.

While predictive models in automated tutoring systems can be successful as teaching tools for specific tasks, P11 warns that student outcomes may not improve if "instead of a teaching tool, it becomes the teacher." In this case, the usage of the automated tutoring system has overstepped the limited intervention scope that was intended to improve outcomes. P11 identifies two main reasons underfunded schools are more susceptible to this overstepping. First, in underfunded schools, administrators or parents might "think the teachers don't know what they're doing, or the teachers are over worked, which they are, and so way more is automated in those environments than should be" (P11). Second, higher income schools often possess the resources to use predictive automated tutoring tools as "part of a balanced breakfast" in tandem with teachers, peers, and other human resources. Successful deployment in high performing or well-resourced schools may lead to "schools in struggling districts [thinking] it's a silver bullet, and it's not used in that same way" (P11). Without the ample teaching resources surrounding the automated tutoring tool, the under-resourced schools end up applying the tutoring tool *in place of* other needful interventions which does not lead to the same learning outcomes for the student.

[*] Name of university withheld to maintain anonymity

In a similar vein to automated tutoring systems, automated grading algorithms can also be susceptible to misuse, with under-resourced classrooms being particularly vulnerable. Deploying grading algorithms for standardized testing can facilitate an extreme version of *teaching to the test*, according to P05. While human graders and rubrics for writing assessments on standardized tests are already formulaic to some extent, automated grading systems are susceptible to strategic gaming that is misaligned with learning goals. P11 comments that in order to appease the automated grader, students may change aspects of their inputs "without understanding why, [...] except that this is what categorizes them as correct versus incorrect." As a specific example, P11 found the automated essay grader for the GRE to be particularly brittle in ways that an English teacher would otherwise likely catch:

> P11: "I remember learning very quickly that if I use transitional phrases, then my score would go way up, even if the content didn't change."

Without adequate teacher support, such usage of an automated grading tool may not help students develop independent writing skills beyond catering to a specific formula.

Since cost-cutting is a powerful driving force behind the adoption of ML4Ed technologies in schools, P05 and P11 worry that students from disadvantaged backgrounds will be disproportionately exposed to technologies that do not serve their learning needs and instead replace teaching under resource constraints. It is therefore an open challenge to address the risk of misuse of ML4Ed technologies, and to design and harness them to bring richer learning experiences to under-resourced students and schools.

C. Additional Discussion of Actionability and Causality. We elaborate on how our findings may be used to extend the framework by Kleinberg et al. (1) to more thoroughly evaluate the practical efficacy of prediction tasks. To illustrate this, consider the following example of a "pure prediction task" from (1): "*The policy challenge is: can we predict which surgeries will be futile using only data available at the time of the surgery? This would allow us save both dollars and disutility for patients.*" The principles from *Translating Predictions to Interventions* show that this example fails to describe the intervention through which prediction would actually save dollars and disutility. Would predicting which surgeries be futile really be the best target here given who will be seeing the predictions? Showing this to doctors could have further externalities of either making doctors overconfident or reducing patient choice (similar to observations in *Harms of Naive Translation from Prediction to Intervention*). Are the inputs mutable or conclusions actionable for the patient (*Need for Actionability (vs. Interpretability)*)? Through these observations and questions, our education study foregrounds a key piece of the translation between predictions and interventions which has previously been taken for granted.

D. Extensions and future work. As our study sheds light on gaps in problem formulation and interventions in ML research, we also highlight a number of open questions in how to further bridge these gaps. For example, given the lack of a clear single objective in education problems shown in *Translating Education Goals to ML Problems*, the formulation of ML problems to navigate the ecosystem of multiple education goals and stakeholders is still a significant open problem. Also, more concrete exploration of how to produce actionable insights using ML would be an impactful avenue of algorithmic study.

There are several extensions to our methodology which could lead to further insights in education and beyond. Interviews with education researchers produce a unique set of discussions which is distinct from interviews with students or teachers. Thus, further fieldwork with direct stakeholders would provide a different and valuable dimension of insight. It may also be illuminating to supplement the qualitative interview study with quantitative statistics from a larger set of applied ML papers. For other domains such as healthcare and criminal justice, this methodology may be applied directly to examine ML research papers and perhaps uncover subtle tensions that were less prevalent in education.

Finally, in the critical evaluation of research practices, another avenue to explore is the degree of cross disciplinary collaboration in applied ML research papers, and relatedly, a comparison of power structures and research incentives in the different fields. Our interviews hinted at the two research fields placing different value judgements for different types of problem formulation, with education researchers trending towards causal interventions and ML researchers preferring predictive modeling. This opens the door for a future exploration of the influence of these competing forces in applied ML research collaborations.

E. Auditing for gaps in educational access. In the context of university admissions, demographic information may be used to audit the existing or proposed admissions process for gaps in education access. ML4Ed papers often report feature weights and other feature importance measures for demographic input features, but these metrics are in general insufficient for assessing whether a proposed algorithmic system is supporting or undercutting "big picture" equity goals. For example, one ML4Ed paper on graduate admissions found that their predictor of admission probability placed zero weight on the demographic attributes of applicants, including gender and ethnicity, and concluded that "admissions decisions are based on academic merit". P06 (PhD candidate), however, points out that this link is tenuous: "it's better than the worst case although we know [gender, ethnicity and national origin] were encoded in other non-demographic features as well." Instead of uncritically interpreting feature weights, participants suggest looking directly at statistics such as student enrollment rates for a realistic picture of education access.

2. Supplementary Materials and Methods

We include the following supplemental information describing our materials and methods, with file names and descriptions of external supplemental files.

1. Demographic and occupational data for interview study participants: Table S1 lists all participants' self-reported occupations, research areas, genders, and races/ethnicities.

2. Pre-interview Survey (SI Dataset S1): This document contains a blank pre-interview survey that was filled out by all interview participants prior to the interview.

3. List of papers referenced in pre-interview survey:

   - SI Dataset S2: This spreadsheet provides a list of all papers presented to interview participants in the pre-interview survey, including title, year of publication, full abstract, conference, paper authors, and education topic keywords (labeled by the authors). The spreadsheet also includes participant responses to indicators of interest and participants who discussed the paper in an interview.
   - Table S2 summarizes the education topic keywords for the 20 papers that were presented in the pre-interview survey as candidates for discussion in interviews.
   - Table S3 summarizes the education topic keywords for the 9 papers that were actually discussed in interviews.

4. Aggregate survey responses (SI Dataset S3): This document contains a summary of participants' responses to selected pre-interview survey questions, including length of time in current position, experiences in the education sector, the number of interview participants who marked each paper as "open to discuss" or "prefer to discuss", as well as aggregate demographic information (gender, race, ethnicity, age range).

5. Interview questions (SI Dataset S4): This document contains a guiding script for the introduction that the interviewers (Author One, Two and Three) presented to the interviewee at the beginning of each interview, as well as guiding questions for the interview.

6. Codes and code frequencies: We provide a full list of all codes used in post-processing the interviews.

   - Figure S1 lists all codes used, as well as the number of interviews in which each code appears.
   - SI Dataset S5: This document provides a list of all codes used in post-processing the interviews with descriptions of each code. This document also contains a complete list of the frequencies of appearance of each code in interviews.

Participant	Occupation	Research area keyword	Gender(s)	Race(s)/ethnicity(ies)
P01	Executive director and full professor	tertiary student success data analytics	M	White
P02	Director of research	economics of education	F	Asian
P03	Associate professor	higher education	F	White
P04	Senior lecturer	education policy	F	White
P05	PhD candidate	college admissions	M	Hispanic and Latino
P06	PhD candidate	higher education	(not reported)	(not reported)
P07	Vice president	higher education	F	White
P08	PhD student	K-12	M	African American
P09	Assistant professor	K-12	M	Asian
P10	Senior director	tertiary student success	M	White
P11	Associate professor	K-12	F	White, MENA/SWANA
P12	Assistant professor	learning at scale	M	White
P13	Full professor	MOOCs	M	Asian
P14	Postdoctoral researcher	education policy	F	Asian, White
P15	PhD candidate	economics of education	F	Hispanic and Latino

Table S1. Summary of study participants. Gender, race, and ethnicity information are voluntarily self-reported.

SI Dataset S1 (pre_survey.pdf)

This document contains a blank pre-interview survey that was filled out by all interview participants prior to the interview.

SI Dataset S2 (list_papers.xlsx)

This spreadsheet provides a list of all papers presented to interview participants in the pre-interview survey, including title, year of publication, full abstract, conference, paper authors, and education topic keywords (labeled by the authors). The spreadsheet also includes participant responses to indicators of interest and participants who discussed the paper in an interview.

Topic keywords	Number of papers
MOOCs	5
Standardized assessment; Psychometrics	4
Language and writing	3
Predicting secondary success; Early warning systems	2
Higher education	2
Course prerequisites and textbooks	2
Student health	2

Table S2. Summary of education topic keywords among the 20 ML4Ed papers that were candidates for interview discussion.

Topic keywords	Number of papers
MOOCs	2
Standardized assessment; Psychometrics	1
Language and writing	1
Predicting secondary success; Early warning systems	2
Higher education	2
Course prerequisites and textbooks	0
Student health	1

Table S3. Summary of education topic keywords among the 9 ML4Ed papers actually discussed in interviews.

SI Dataset S3 (Aggregate_survey_responses.docx)

This document contains a summary of participants' responses to selected pre-interview survey questions, including length of time in current position, experiences in the education sector, the number of interview participants who marked each paper as "open to discuss" or "prefer to discuss", as well as aggregate demographic information (gender, race, ethnicity, age range).

SI Dataset S4 (Interview_questions.pdf)

This document contains a guiding script for the introduction that the interviewers (authors one, two, and three) presented to the interviewee at the beginning of each interview, as well as guiding questions for the interview.

A. Codes and code frequencies. We provide a full list of all codes used in post-processing the interviews. Figure S1 lists all codes used, as well as the number of interviews in which each code appears. We also provide a more detailed breakdown of code frequencies per interview in the attached dataset S5.

SI Dataset S5 (codes_and_frequencies.xlsx)

This document provides a list of all codes used in post-processing the interviews with descriptions of each code. This document also contains a complete list of the frequencies of appearance of each code in each participant's interview.

References

1. J Kleinberg, J Ludwig, S Mullainathan, Z Obermeyer, Prediction policy problems. *Am. Econ. Rev.* **105**, 491–95 (2015).

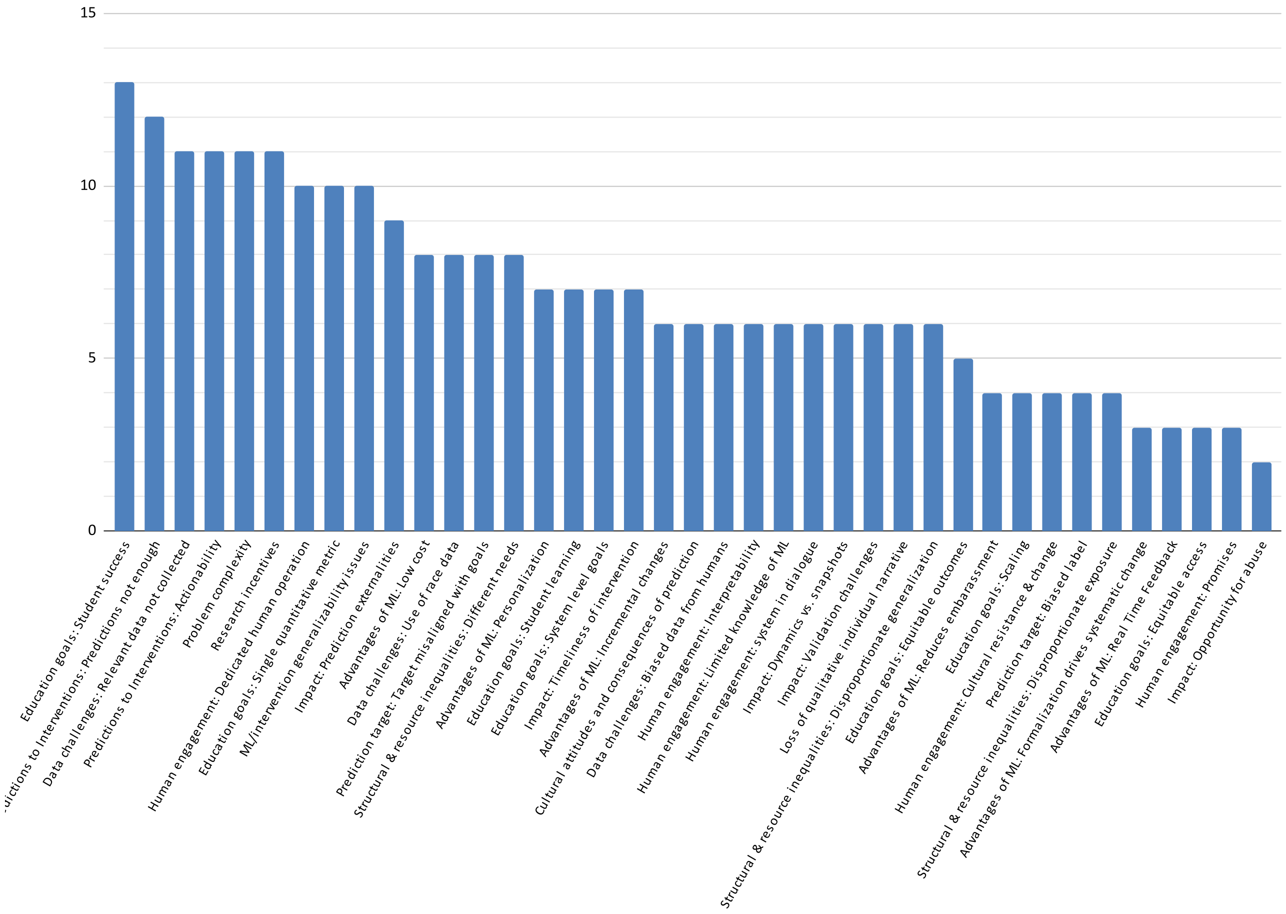

Fig. S1. List of codes and number of interviews in which each code appears.
