# Supplementary material for: Reimagining the machine learning life cycle to improve educational outcomes of students
Source: Proc Natl Acad Sci U S A. 2023 Feb 24;120(9):e2204781120. doi: 10.1073/pnas.2204781120 (PMC9992853; doi:10.1073/pnas.2204781120)
Supplement: Supplementary file 2 — Dataset S01 (PDF) [file pnas.2204781120.sd01.pdf]

Pre-interview Survey on Data Science and Machine Learning for Education

This is a pre-interview survey for an upcoming interview. In this survey, we ask for information that will help us select material for this interview.

Our goal for the interview is to understand how machine learning is impacting the education sphere by talking to education experts. We would like to evaluate the extent to which current machine learning algorithms are able to capture the objectives of equity and justice in education. We would like to critically examine -- even in cases where the project is designated as being "machine learning for social good" -- whether these algorithms capture the societal goals and what impact they might have on educational inequalities.

We're generally motivated by the following questions:

1. In the places where data science and machine learning are currently being applied in the education sphere, is the setup of the machine learning task aligned with the intended real impact on students?
2. Are there areas where the use of data science and machine learning is unintentionally hurting students? What about areas where data science and machine learning is helping students?
3. What improvements can we make to machine learning systems to better serve students (e.g. improvements on data, success metrics, or machine learning problem formulation)?

To guide our discussion, we have prepared a few examples of papers and contests where data science and machine learning are applied with the goal of helping students. For each example, we will outline the societal/educational goal, and the machine learning task (including the data and the labels). Part of this survey will ask you to indicate which of these papers best matches your expertise.

---

* Required

1. Email *

____

2. Name *

____

3. What is your current position? *

e.g., assistant professor, associate professor, full professor, graduate student, research scientist, policy-maker, other practitioner etc.

4. How long have you been in this position? *

5. What experiences have you had in the educational sector? (Select all that apply)

Check all that apply.

- [ ] taught at an elementary/middle/high school
- [ ] taught at a university
- [ ] worked with not-for-profit organization(s) or NGO (excluding not-for-profit K-12 or higher ed institution
- [ ] worked with for-profit organizations in education
- [ ] worked with policymakers
- [ ] created or designed curriculum
- [ ] built tools used in the education sector

Other: [ ]

6. If you'd like, please elaborate on any of the experiences above (e.g. specific organizations you worked with, specific tools, etc.)

(Optional)

7. Please provide 2-3 keywords that describe your research areas.

e.g., curriculum, education policy, evaluation, history, language and literacy, organization, philosophy, psychology, technol international education, school counseling, special education, race, gender, teachers and teacher learning, mathematics education, science education, right to education, early childhood, MOOCs, learning management systems, etc.

Selection of papers	To guide our discussion, we've prepared examples of papers and contests where data science and machine learning is applied to education. We have prepared two types of material to review:<br><br>1. Papers on applications of data science and machine learning to education.<br>2. Previous data science and machine learning contests with applications to education (e.g. contests from computer science conferences such as NeurIPS and KDD).<br><br>In the interview, we will discuss one contest and one paper, according to your interest and expertise.<br><br>NOTE ON CONFLICTS OF INTEREST: Please do not select any papers or contests where you have an individual conflict of interest with any of the authors.<br><br>The following constitutes an individual conflict of interest:<br><br>- Family relationship or close personal relationship<br>- Graduate advisee/advisor relationship<br>- Any current, recent, or recurring collaboration (including grants and internships) ("recent" means "within the last three years.")

Please select UP TO SIX of the following papers that you would prefer to discuss or are open to discuss (leave the checkboxes blank otherwise). We will aim to select papers that you indicate you prefer to discuss. The papers are roughly organized by broad categories below. Abstracts and details of each paper can be found here: https://docs.google.com/spreadsheets/d/1LID5Pc8dsj_pN-jUDaH07_djRYNk6ab3pncSyNLT6S4/edit?usp=sharing

8. Standardized assessment; Psychometrics

Check all that apply.

	Open to discuss	Prefer to discuss
1. Time-varying learning and content analytics via sparse factor analysis	☐	☐
2. Question Difficulty Prediction for READING Problems in Standard Tests	☐	☐
3. QuesNet: A Unified Representation for Heterogeneous Test Questions	☐	☐
4. Knowledge Tracing Machines: Factorization Machines for Knowledge Tracing	☐	☐

9. MOOCs

Check all that apply.

	Open to discuss	Prefer to discuss
5. Probabilistic Graphical Models for Boosting Cardinal and Ordinal Peer Grading in MOOCs	☐	☐
6. Learning Latent Engagement Patterns of Students in Online Courses	☐	☐
7. Identifying At-Risk Students in Massive Open Online Courses	☐	☐
8. Deep Reinforcement Learning for Syntactic Error Repair in Student Programs	☐	☐
9. Zero Shot Learning for Code Education: Rubric Sampling with Deep Learning Inference	☐	☐

10. Predicting college or secondary success; Early warning systems

Check all that apply.

	Open to discuss	Prefer to discuss
10. A Machine Learning Framework to Identify Students at Risk of Adverse Academic Outcomes	☐	☐
11. Predicting student risks through longitudinal analysis	☐	☐

11. Higher education

Check all that apply.

	Open to discuss	Prefer to discuss
12. Progressive Prediction of Student Performance in College Programs	☐	☐
13. GRADE: Machine Learning Support for Graduate Admissions	☐	☐

12. Language and writing

Check all that apply.

	Open to discuss	Prefer to discuss
14. Spoken English Grading: Machine Learning with Crowd Intelligence	☐	☐
15. A Semantics-based Model for Predicting Children's Vocabulary	☐	☐
16. Formative Essay Feedback Using Predictive Scoring Models	☐	☐

13. Course prerequisites and textbooks

Check all that apply.

	Open to discuss	Prefer to discuss
17. Semi-Supervised Techniques for Mining Learning Outcomes and Prerequisites	☐	☐
18. Inferring Concept Prerequisite Relations from Online Educational Resources	☐	☐

14. Student health

Check all that apply.

	Open to discuss	Prefer to discuss
19. Probabilistic Latent Variable Modeling for Assessing Behavioral Influences on Well-Being	☐	☐
20. Exercise-Enhanced Sequential Modeling for Student Performance Prediction	☐	☐

15. Confirmation of lack of conflicts of interest *

Check all that apply.

☐ I confirm that I do not have any known individual conflict of interest with the authors of the papers I selected. The following constitutes an individual conflict of interest: Family relationship or close persona relationship; Graduate advisee/advisor relationship; Any current, recent, or recurring collaboration (includ grants and internships) ("recent" means "within the last three years.")

Demographic Information

This section is optiona

16. Which gender(s) do you identify with?

Check all that apply.

☐ Female
☐ Male
☐ Genderqueer/non-binary
☐ Prefer not to say
Other: ☐ ______________________

17. Which race(s) or ethnicity(ies) do you identify with?

Check all that apply.

- [ ] African American
- [ ] American Indian or Alaska Native
- [ ] Asian
- [ ] Black
- [ ] Hispanic and Latino
- [ ] Native Hawaiian or Other Pacific Islander
- [ ] White
- [ ] Prefer not to say

Other: [ ] ______________________

18. What is your age range?

Mark only one oval.

- ( ) 18 to 24 years
- ( ) 25 to 44 years
- ( ) 45 to 64 years
- ( ) 65 years and over
- ( ) Prefer not to say

Interview Scheduling

19. If we have not yet scheduled a time for the interview, please sign up for an hour-long intervi slot at this link: https://calendly.com/mlforeducation2021/interview

Mark only one oval.

- ( ) I have signed up for a time.
- ( ) There were no times that worked for me.
- ( ) We already have previously scheduled a time.
- ( ) Other: ______________________

20. Before the interview, we kindly ask that you read and sign an Online Consent Form for conse to participate in our study. This should have been sent by email over DocuSign along with the pre-interview survey. Please select the option below that applies:

Mark only one oval.

- ( ) I have received the Online Consent Form.
- ( ) I have not received the Online Consent Form yet.

This content is neither created nor endorsed by Google.

Google Forms
