# Supplementary material for: Reimagining the machine learning life cycle to improve educational outcomes of students
Source: Proc Natl Acad Sci U S A. 2023 Feb 24;120(9):e2204781120. doi: 10.1073/pnas.2204781120 (PMC9992853; doi:10.1073/pnas.2204781120)

Aggregate Survey Responses

Q: What is your current position?

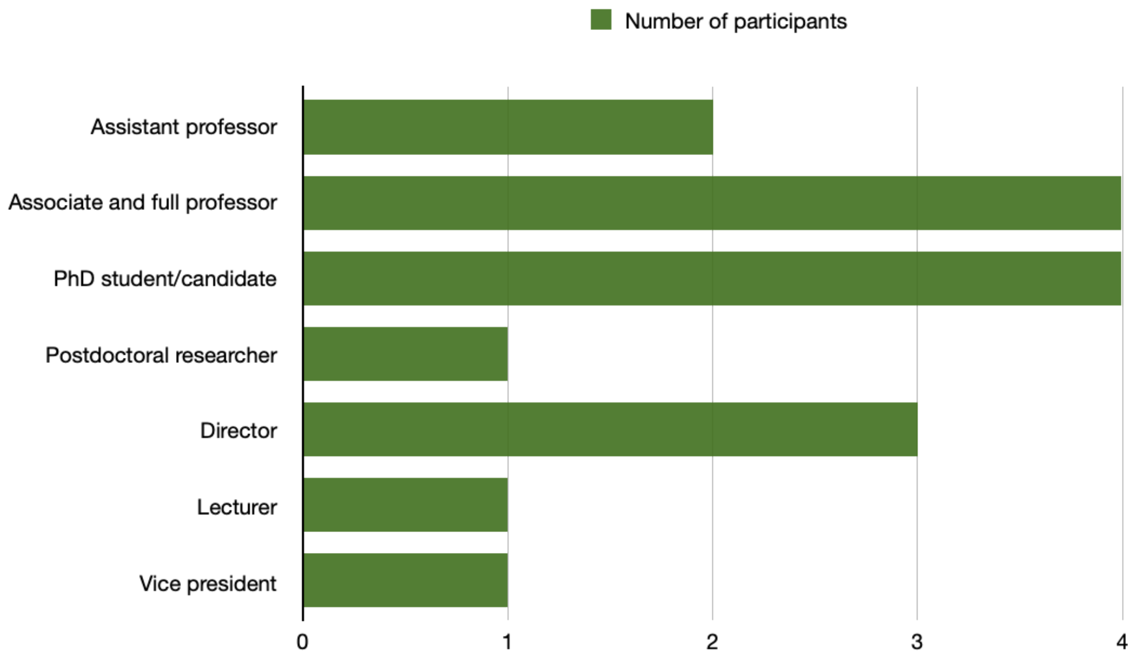

Q: How long have you been in this position?

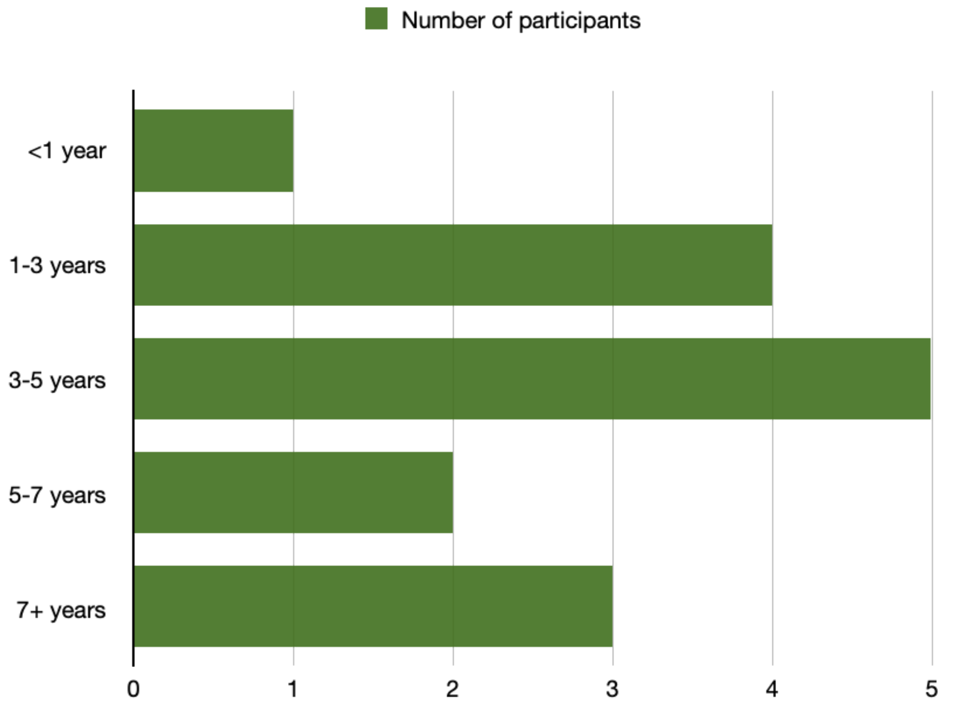

Q: What experiences have you had in the educational sector? (Select all that apply)

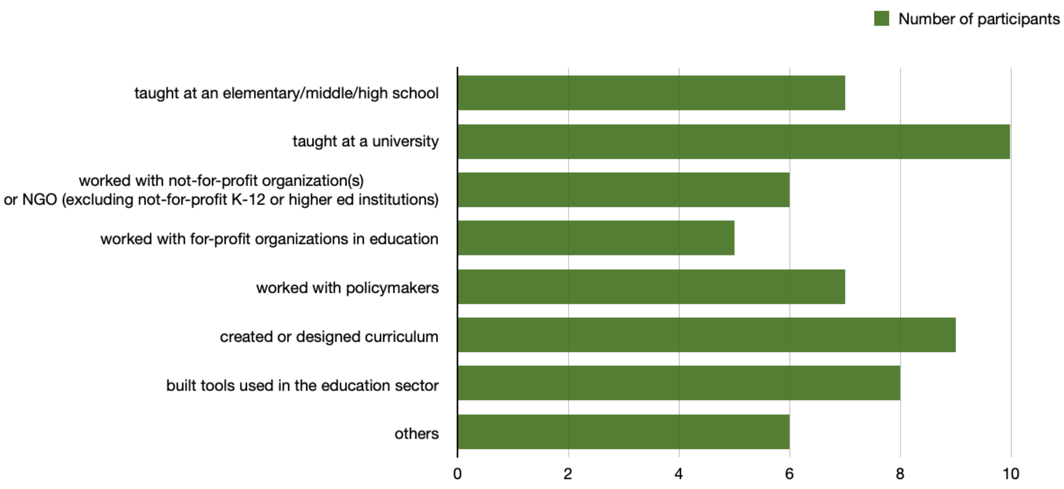

Q: “Please select UP TO SIX of the following papers that you would prefer to discuss or are open to discuss (leave the checkboxes blank otherwise). We will aim to select papers that you indicate you prefer to discuss. The papers are roughly organized by broad categories below. ”

No.	Title	Open to discuss (Number of participants)	Prefer to discuss (Number of participants)
	**Standardized assessment; Psychometrics**		
1	Time-varying learning and content analytics via sparse factor analysis	1	0
2	Question Difficulty Prediction for READING Problems in Standard Tests	1	0
3	QuesNet: A Unified Representation for Heterogeneous Test Questions	0	0
4	Knowledge Tracing Machines: Factorization Machines for Knowledge Tracing	0	0
	**MOOCs**		
5	Probabilistic Graphical Models for Boosting Cardinal and Ordinal Peer Grading in MOOCs	1	0

6	Learning Latent Engagement Patterns of Students in Online Courses	2	2
7	Identifying At-Risk Students in Massive Open Online Courses	5	2
8	Deep Reinforcement Learning for Syntactic Error Repair in Student Programs	0	0
9	Zero Shot Learning for Code Education: Rubric Sampling with Deep Learning Inference	0	1
	**Predicting secondary success; Early warning systems**		
10	A Machine Learning Framework to Identify Students at Risk of Adverse Academic Outcomes	5	7
11	Predicting student risks through longitudinal analysis	6	7
	**Higher education**		
12	Progressive Prediction of Student Performance in College Programs	7	5
13	GRADE: Machine Learning Support for Graduate Admissions	5	5
	**Language and writing**		
14	Spoken English Grading: Machine Learning with Crowd Intelligence	2	1
15	A Semantics-based Model for Predicting Children's Vocabulary	1	1
16	Formative Essay Feedback Using Predictive Scoring Models	1	4
	**Course prerequisites and textbooks**		
17	Semi-Supervised Techniques for Mining Learning Outcomes and Prerequisites	4	1

18	Inferring Concept Prerequisite Relations from Online Educational Resources	2	0
	**Student health**		
19	Probabilistic Latent Variable Modeling for Assessing Behavioral Influences on Well-Being	2	1
20	Exercise-Enhanced Sequential Modeling for Student Performance Prediction	0	0

Demographic Information

Q: Which gender(s) do you identify with?

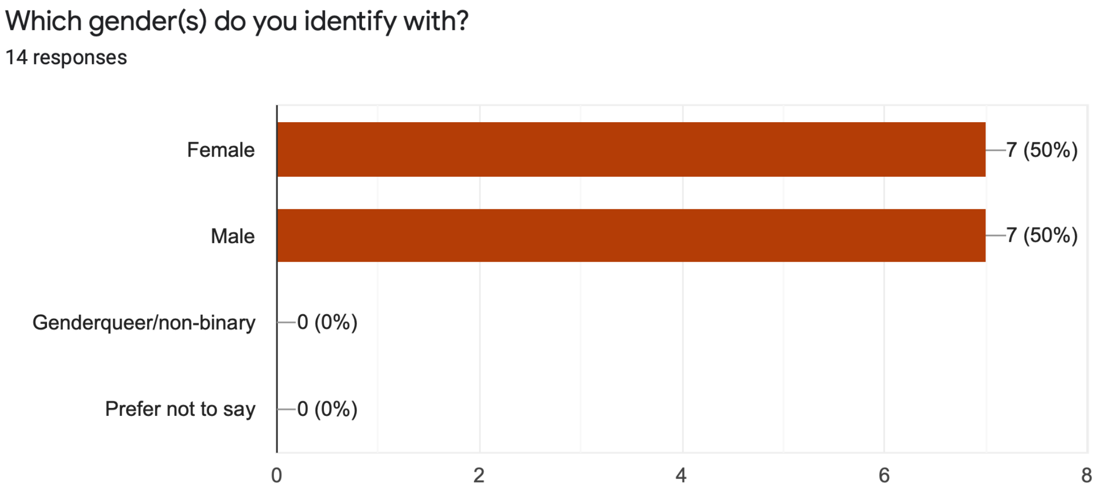

Q: Which race(s) or ethnicity(ies) do you identify with?

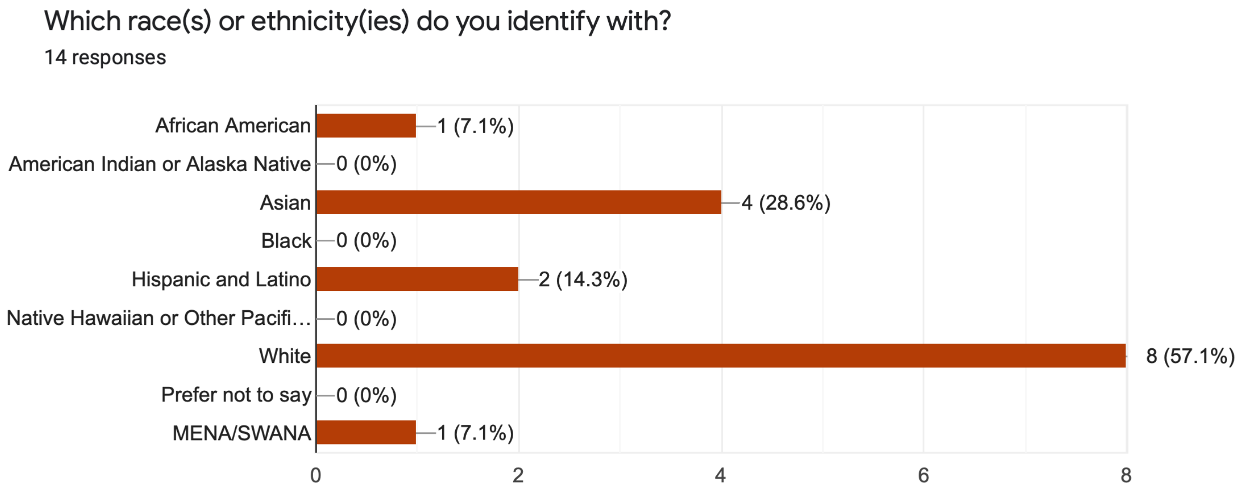

Q: What is your age range?

What is your age range?

14 responses

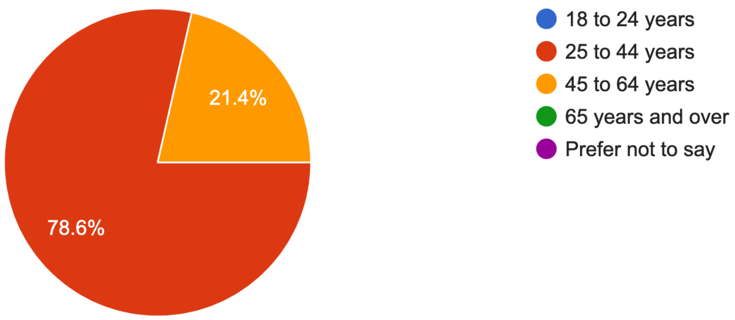

Supplement: Supplementary file 4 — Dataset S03 (PDF) [file pnas.2204781120.sd03.pdf]
