# Supplementary material for: Reimagining the machine learning life cycle to improve educational outcomes of students
Source: Proc Natl Acad Sci U S A. 2023 Feb 24;120(9):e2204781120. doi: 10.1073/pnas.2204781120 (PMC9992853; doi:10.1073/pnas.2204781120)
Supplement: Supplementary file 5 — Dataset S04 (PDF) [file pnas.2204781120.sd04.pdf]

Interview Questions

Introduction

We are interested in problems of equity and justice in education. We're trained in computer science, so while we are familiar with the computational aspects of algorithms, we see a growing gap in our field between applying technical solutions and deeply engaging with the real life problems of equity and justice in education.

These days, more and more machine learning algorithms are being deployed in the education sector that impact students, and our goal is to evaluate the extent to which current machine learning algorithms are able to capture the real societal objectives of equity and justice in education. We would like to critically examine -- even in cases where the project is designated as "machine learning for social good" -- whether these algorithms are doing a good job, or being deployed naively in ways that could even reinforce inequalities. We're hoping that by talking to you, we'll gain a better picture of how machine learning algorithms are helping or hurting real students.

To guide our discussion, we've prepared a few examples of papers where machine learning is applied with the goal of helping students. For each example, we will outline the societal/educational goal, and the machine learning task (including the data and the labels).

Part 1: introductions and general questions

- What are your current research interests in education? In your survey response, you mentioned that you were interested in …, could you tell us more about that?
- What examples can you think of that use data science or machine learning in education contexts?
    - In which of these examples do you see machine learning making a positive / negative impact on students?

Part 2: deep dive into contests and papers

- Walking through a section: How would you describe their goals?
- To what extent do you feel that {societal/educational goal} is a problem in education?
    - Are there people in education who work on this?
    - What attempts to solve this problem are you aware of?
- To what extent do you feel these {label/objective functions} capture the {societal/educational goal}?
    - If good:
        - Why do you think it's good? What metrics by researchers in education and are they aligned?

        - Would you anticipate heterogeneous effects from this proposal?
    - If neutral/bad:
        - What does it seem to be capturing well/ok?
        - What is it missing?
        - Do you have suggestions for proposals that would mitigate this?
        - Do you think there could be student groups harmed? If so, which student groups are helped/harmed by this approach?
- Findings:
    - To what extent do these mirror what you'd expected?
    - Is this in line with findings in the education literature?
    - How do think these findings could be used in education?
